# Time dependent predictors of cardiac inflammatory adverse events in cancer patients receiving immune checkpoint inhibitors

**DOI:** 10.1186/s40959-025-00331-8

**Published:** 2025-04-28

**Authors:** Michael Sayer, Hirofumi Hamano, Misako Nagasaka, Benjamin J. Lee, Jean Doh, Pranav M. Patel, Yoshito Zamami, Aya F. Ozaki

**Affiliations:** 1 School of Pharmacy & Pharmaceutical Sciences, University of California, 802 W Peltason Dr, Room 106A, Irvine, CA 92617 USA; 2 Department of Pharmacy, Okayama University Hospital, Okayama, Japan; 3 Division of Hematology and Oncology, University of California, Irvine, CA USA; 4 Department of Pharmacy, University of California Irvine Health, Orange, CA USA; 5 Division of Cardiology, Department of Medicine, University of California, Irvine, CA USA

**Keywords:** Immune checkpoint inhibitors, Immune-Related adverse events, Myocarditis, Pericarditis, Predictive modeling, TriNetx

## Abstract

**Background:**

Cardio-inflammatory immune related adverse events (irAEs) while receiving immune checkpoint inhibitor (ICI) therapy are particularly consequential due to their associations with poorer treatment outcomes. Evaluation of predictive factors of these serious irAEs with a time dependent approach allows better understanding of patients most at risk.

**Objective:**

To identify different elements of patient data that are significant predictors of early and late-onset or delayed cardio-inflammatory irAEs through various predictive modeling strategies.

**Methods:**

A cohort of patients receiving ICI therapy from January 1, 2010 to May 1, 2022 was identified from TriNetX meeting inclusion/exclusion criteria. Patient data collected included occurrence of early and later cardio-inflammatory irAEs, patient survival time, patient demographic information, ICI therapies, comorbidities, and medication histories. Predictive and statistical modeling approaches identified unique risk factors for early and later developing cardio-inflammatory irAEs.

**Results:**

A cohort of 66,068 patients on ICI therapy were identified in the TriNetX platform; 193 (0.30%) experienced early cardio-inflammatory irAEs and 175 (0.26%) experienced later cardio-inflammatory irAEs. Significant predictors for early irAEs included: anti-PD-1 therapy at index, combination ICI therapy at index, and history of peripheral vascular disease. Significant predictors for later irAEs included: a history of myocarditis and/or pericarditis, cerebrovascular disease, and history of non-steroidal anti-inflammatory medication use.

**Conclusions:**

Cardio-inflammatory irAEs can be divided into clinically meaningful categories of early and late based on time since initiation of ICI therapy. Considering distinct risk factors for early-onset and late-onset events may allow for more effective patient monitoring and risk assessment.

**Supplementary Information:**

The online version contains supplementary material available at 10.1186/s40959-025-00331-8.

## Introduction

Immune checkpoint inhibitors (ICIs) have significantly improved survival outcomes in cancer patients although its utilization has been associated with a unique class of toxicities. Immune-related adverse events (irAEs) from ICI therapy have wide ranging effects on the body often caused by excessive inflammation stimulated by unchecked immune system activity [1]. Cardiac inflammation, characterized as myocarditis or pericarditis, is a rare irAE occurring in approximately 1% of patients receiving ICI therapy [2, 3]. While some types of irAEs like thyroiditis and dermatitis can have positive survival implications and are readily managed, cardiac irAEs are associated with a dire prognosis including hospitalization for their management and an increased mortality risk [4, 5]. Discontinuing ICI therapy is one approach to management, however this can prevent patients from receiving optimal cancer treatment [6]. The most common intervention is treatment with corticosteroids, which also my limit the efficacy of ICI agents due to immunosuppression [7]. Established labs and tests like troponin levels or ECG readings that might identify people at risk or allow for patient monitoring are not routinely available for a majority of ICI patients, especially those receiving therapy in the outpatient setting over many months [8–10]. To this point, some preliminary risk factors for the development of cardio-inflammatory adverse events identified include ICI combination therapy, biological sex, pre-existing cardiovascular comorbidities, and select medications [11–13]. Given limited monitoring ability and effective interventions, identification of time-dependent risk factors may allow for better identification of patients who are at risk for these serious irAEs over time.

While the timing of adverse events for other oncology agents appears more predictable, cardiac irAE onset can occur after single infusions of ICI therapy or more than 1 year removed from therapy initiation consistent with irAEs impacting other body systems [14–16]. Identifying distinct risk factors for early versus later adverse events may be warranted for a variety of reasons. With more common irAEs like dermatitis or thyroiditis, the risk factors for their occurrence can be significantly influenced by a patients time on ICI therapy [17–21]. Additionally, with certation irAEs like pneumonitis and hepatitis, earlier onset events are associated with poorer overall and progression free survival outcomes compared to later onset events [22, 23]. Preliminary studies suggest distinct physiological and immune pathways associated with irAEs dependent on their timing, with later events more likely to be triggered by dysregulated humoral immune response while early onset events associated with cell-mediated immunity [24, 25]. Lastly, cancer patient populations can vary significantly in their treatment trajectories over time. Patients who experience mortality outcomes within a few months of initiating ICI therapy are likely to have distinct cancer severities, demographic backgrounds, or medical histories making risk factors for cardiac irAEs evolve as treatment progresses. Given these considerations, exploring time-dependent risk factors for cardiac irAEs is warranted.

Literature assessing cardiac inflammatory adverse events predominately focuses on myocarditis, leading to limited generalizability of ICI impact on cardiovascular health. Consideration of pericarditis may provide a more complete picture, with preliminary studies suggesting that pericarditis irAEs lead to increased mortality rates similar to myocarditis [26]. Similar causative pathways and significant relationships between ICI induced inflammation of pericardial tissue and myocarditis have also been identified [27]. Considering myocarditis and pericarditis as cardiac inflammatory adverse events together will allow for more nuanced analysis and better capture the impact of ICIs on cardiovascular health. A challenge in assessing risk factors for cardiac inflammation in ICI patients, especially in a time dependent manner, is having a representative sample [28]. TriNetX is a unique repository of real-world healthcare data with a network of hundreds of health care institutions, allowing for the creation of unique large cohorts of patients receiving ICI therapies [29]. Contemporary studies investigating cardio-inflammatory irAEs feature smaller samples ranging from a select few cases to rarely exceeding 100 total patients [30–32]. Leveraging the TriNetX platform, uniquely large cohorts of ICI patients can be utilized to investigate predictive factors of early and later cardio-inflammatory irAEs. Considering myocarditis and pericarditis as cardio-inflammatory irAEs together, in addition to distinguishing between early and later onset events, may allow for identification of novel risk factors predisposing patients to them.

## Methods

### Study design and data source

We performed a multicenter, multinational electronic health research retrospective cohort study, evaluating all patients receiving ICI therapy within the TriNetX platform from 80 international healthcare institutions providing the data. Files of patient data were obtained on May 15, 2023, including reported encounters, diagnoses, and medications received. Using these files, events occurring after ICI administration and relevant medical histories prior were obtained for subsequent analyses.

### Patient population

Medical records for patients receiving ICI therapies from January 1, 2010 to May 1, 2022 were queried from the TriNetX platform. Inclusion criteria for the study were: adult patients 18 years and older at time of ICI initiation, with a history of neoplasm diagnosis, having birthdate and biological sex information, and survival times consistent with ICI initiation times (Supplemental Table 1). Patients with myocarditis/pericarditis diagnoses up to 6 months prior to ICI initiation were excluded from the patient population; this prevented pre-existing conditions from being counted as cardiac adverse events (Supplemental Table 1). Survival times were utilized in selecting appropriate patient populations for prospective analyses. These were defined as the number of days since from ICI therapy initiation until patients were either deceased or until there last encounter in the database.

### Index immune checkpoint inhibitor(s) and index date

Index ICI’s for each prospective patient were defined as the agent(s) given for their first administration of ICI therapy. Index ICI’s considered were: anti-PD-1 monotherapy (i.e., nivolumab, pembrolizumab), anti-PD-L1 monotherapy (i.e., atezolizumab), and Combination ICI therapy. The index dates utilized in subsequent analyses were the reported date of administration of a patient’s first ICI therapy (Supplemental Table 1).

### Cardiac inflammation adverse events

Cardiac inflammation adverse events were defined by International Classification of Diseases, Ninth (ICD-9) and Tenth (ICD-10) Revision codes representing both myocarditis or pericarditis consistent with prior studies (Supplemental Table 2) [33–35]. Adverse events within the study were defined as the occurrence of selected diagnosis codes within a patient’s medical record on or within 365 days after ICI index date. Time to adverse event was defined as the number days removed from ICI index date a patient experienced an event. Early adverse events were defined as occurring within 90 days of index date, while later events were defined as occurring after consistent with other studies [24, 36–38]. We respect that “early” and “late” onset event definitions can be subjective with other studies defining them based on other factors like number of cycles received (1 or 2 cycles) or shorter thresholds relative to initiation (i.e. 6 weeks). However, we feel our definition is supported and sufficiently distinguishes acute and chronic exposures to ICI agents. Patients were considered to have a history of cardiac inflammation if they experienced relevant diagnosis codes more than 6 months prior to ICI therapy initiation (patients within 6 months were excluded from the study).

### Covariates utilized

A variety factors ranging from patient demographic information, history of comorbidities, and medication history were considered as predictors of cardiac inflammation adverse events (Supplementary Tables 1&2). Age at index was the number of years from date of birth to index ICI date. Due to incomplete reporting on racial and ethnic backgrounds of patients and limited sample sizes, patients were dichotomized as “Non-Hispanic White/Caucasian” or “Non-White/Caucasian.” Patients were considered to have a history of relevant comorbidities if they occurred within 5 years prior to their index date. Specific comorbidities were selected based on their relevance to cardiovascular health. Relevant ICD-9 and 10 codes were selected based on previous work identifying relevant codes to assess patient medical history in insurance claims (Supplemental Table 2) [39, 40]. Patients were considered as having a history use of selected medications or medication classes if they had documented of them up to 100 days prior to their index date (Supplemental Table 1). Select medications were selected if they were generally commonly used and either having cardiovascular indications or have proven associations with increased cardiac inflammation. Descriptive statistics were utilized to describe the prevalence of these features within the entire population, within patients experiencing early events, and within patients experiencing later adverse events.

### Logistic regression modeling

Separate logistic regression models were implemented predicting cardiac inflammatory adverse events occurring within 90 days of index date and later adverse events. Models predicting early events included all patients that experienced adverse events and patients having survival times of at least 90 days. Models predicting later events excluded patients experiencing early adverse events and utilized patients having survival times of at least 365 days (Supplemental Fig. 2). Univariate logistic regression analyses were implemented to identify predictors for the occurrence of early and later adverse events. Variables having a *P*-value less than 0.1 were selected for consideration into multivariate models. These selected variables were utilized in separate baseline logistic regression models predicting early and later adverse events. Following creation of baseline models, Akaike information criterion (AIC) optimized models were created utilizing an exhaustive algorithm starting with the created baseline models. Significance of individual feature(s) for each prospective logistic regression model were assessed based on Wald statistics and their associated *P*-values with a significance threshold of 0.05. Variance inflation factors (VIF) were calculated to assess multicollinearity in multi-variable models, with a threshold value of 5 utilized.

### Cox-proportional hazards modeling

Cox Proportional Hazards (COXPH) models were utilized to determine hazard ratios for variables identified with univariate feature selection relevant to occurrence of early and later adverse events. The COXPH model for early adverse events included all patients meeting initial inclusion/exclusion criteria, while the COXPH model for later adverse events excluded these patients experiencing early adverse events and only included patients having at least 90 days of survival (Supplemental Fig. 2). Significance of individual features of COXPH models were assessed based on Wald statistics and their associated *P*-values with a significance threshold of 0.05. Censoring due to morality and follow up time month over month was determined. Schoenfeld residuals were evaluated for early and late onset cox-models to ensure appropriate use of this strategy. Variance inflation factors (VIF) were calculated to assess multicollinearity, with a threshold value of 5 utilized.

Various steps in data analysis were conducted in the R-programing platform version 4.1.1. Data cleaning and creation of descriptive statistics utilized base R-language and the DPLYR package. The comorbidity package within R aided in assessment of patient comorbidity history. Logistic regression models were created with the stats, bestglm, and caret packages. Lastly, we utilized survival and survminor packages to create COXPH models.

## Results

### Patient population

A total of 66,068 patients receiving ICI therapy were identified from the TriNetX database meeting study inclusion/exclusion criteria (Supplemental Fig. 1). A total of 368 (0.56%) patients experienced cardiac inflammatory adverse events, with 193 (0.30%) experiencing early adverse events, 175 (0.26%) experiencing later adverse events, and 135 (0.20%) patients having a history of relevant myocarditis/pericarditis diagnoses in their medical history (Table 1). The average patient age at index was 65 years of age, 76.8% of patients were identified as Caucasian or White, and 57.4% of patients were male. ICI therapy utilized at index was an anti-PD-1 agent for 75.1% of patients, a anti-PD-L1 agent in 14.5% of patients, and a combination ICI therapy in 7.0% of patients (Table 1). Prevalence of different patient characteristics of interest included: history myocardial infarction (MI) in 6.7%, congestive heart failure (CHF) in 9.6%, peripheral vascular disease (PVD) in 18.4%, cerebrovascular disease (CEVD) in 11.9%, hypertension (HTN) in 49.0%, and diabetes mellitus (DM) in 19.3% of patients (Table 1). Medication use histories are also reported (Table 1). Additionally, the distribution of these characteristics amongst patients not experiencing a cardio-inflammatory irAE, patients experiencing an early adverse event, and patients experiencing later events are also reported (Supplemental Table 3).

**Table 1:** Descriptive statics for immune checkpoint inhibitor patient population

Variable Category	Variable Name	Count *N* = 66,068	Percentage/ Average
Total Adverse Events	*Adverse Events*	368	0.56
	*Early Adverse Events*	193	0.29
	*Later Adverse Events*	175	0.26
	*Myocarditis/Pericarditis History*	135	0.2
	*Age at Index*	NA	65
	*Male*	37,946	57.43
	*Caucasian*	50,751	76.82
Index ICI	*Anti-PD-1*	49,668	75.18
	*Anti-PD-L1*	9606	14.54
	*Combo*	4598	6.96
Comorbidities	*Myocardial Infarction*	4448	6.73
	*Congestive Heart Failure*	6328	9.58
	*Peripheral Vascular Disease*	12,143	18.38
	*Cerebrovascular Disease*	7873	11.92
	*Hypertension*	32,352	48.97
Medication History	*Diabetes Mellitus*	12,640	19.13
	*ACE-I*	6329	9.58
	*ARB*	4636	7.02
	*Beta-Blocker*	13,950	21.11
	*Calcium Channel Blocker*	8712	13.19
	*Thiazide*	4392	6.65
	*Loop Diuretic*	6868	10.4
	*Aldosterone Antagonist*	1708	2.59
	*Statin*	12,259	18.56
	*Aspirin*	8350	12.64
	*Anti-Platelet*	1627	2.46
	*Anti-Coagulant*	32,717	49.52
	*Metformin*	2959	4.48
	*Dipeptidyl peptidase 4 inhibitors*	648	0.98
	*Sodium-Glucose Transport Protein 2 inhibitors*	337	0.51
	*Insulin*	6543	9.9
	*Atypical Antipsychotic*	2472	3.74
	*Sulfonamide*	2782	4.21
	*Non-steroidal Anti-Inflammatory*	12,648	19.14

The first column from left to right represents the variable category, variable name, the total number of patients, and the percentage or average of the total study population. Categorical data are reported as percentages within the patient population while numeric variables have reported as averages

### Univariate logistic regression analysis and feature selection for multivariate models predicting early and later cardiac inflammatory adverse events

The following variables had significant associations with the occurrence of early cardiac inflammatory adverse events in univariate analysis: anti-PD-L1 agent at index, combination ICI therapy at index, prior history of CHF, history of PVD, history of CEVD, history of HTN, history of aspirin, history of anti-coagulant, and history of atypical antipsychotic (Supplemental Table 4). No additional variables met the threshold of significance having a *P*-value less than 0.1 for inclusion in multivariate models predicting early adverse events (Supplemental Table 4). Variables having a significant association with development of later adverse events included: history of diagnosis myocarditis and/or pericarditis, history of CHF, history of CEVD, history of HTN, history of DM, history of ACE-Inhibitor (ACE-I), history of beta-blocker, statin, and non-steroidal anti-inflammatory (NSAID) use (Supplemental Table 4). The following features met the threshold for inclusion in multivariate models predicting later adverse events with *P*-values less than 0.1: combination therapy at index, history of loop diuretic, and history of anti-coagulant (Supplemental Table 4).

### Baseline and optimized multi-variate regression analyses predicting early and later adverse events

Within the baseline logistic regression models predicting early adverse events, the following variables were significant: combination ICI therapy (OR:4.66, 95% CI = 2.79–7.99) at index and a history of peripheral vascular disease (OR:1.54, 95% CI = 1.09–2.15). The optimized logistic regression model predicting early events had 2 significant features including combination therapy at index (OR:4.67, 95% CI = 2.79–7.99) and history of peripheral vascular disease (OR:1.71, 95% CI = 1.23–2.34) (Table 2). The optimized model additionally kept anti-PD-1 agent at index (OR:1.48, 95% CI = 0.97–2.37), history of anticoagulant (OR:1.30, 95% CI = 0.97–1.75) and history of atypical antipsychotic (OR:1.70, 95% CI = 0.86- 3.00) as predictors although they were not significant (Table 2). No features had variance inflation factors above 5, indicating no issues with multi-collinearity.

**Table 2:** Coefficients and *P*-values for baseline and optimized logistic regression models predicting early and later cardio-inflammatory IrAEs

Variable	Baseline 3-Months		Optimized 3 Months		Baseline 3–12 Months		Optimized 3–12 Months	
	HR (95%CI)	coefficient (*p*-value)	HR (95%CI)	coefficient (*p*-value)	HR (95%CI)	coefficient (*p*-value)	HR (95%CI)	coefficient (*p*-value)
**Myocarditis/Pericarditis History** *Binary Variable (ref = No)*					8.19 (1.95–23.10)	2.104 (0.0005)****	8.67 (2.08- 24.00)	2.16 (0.0003)****
**Anti-PD-1 Agent** *Binary Variable (Yes or No)*	1.49 (0.97–2.34)	0.396 (0.081)*	1.48 (0.97–2.37)	0.395 (0.081)*				
**Combination Therapy** *Binary Variable (ref = No)*	4.66 (2.79–7.99)	1.541 (7.85e-9)****	4.67 (2.79–7.99)	1.54 (7.78e-9)****	1.56 (0.93–2.45)	0.444 (0.069)*	1.54 (0.92–2.43)	0.436 (0.075)*
**Congestive Heart Failure** *Binary Variable (ref = No)*	1.14 (0.71–1.75)	0.128 (0.577)			1.15 (0.67–1.86)	0.138 (0.589)		
**Peripheral Vascular Disease** *Binary Variable (ref = No)*	1.54 (1.09–2.15)	0.432 (0.013)**	1.71 (1.23–2.34)	0.538 (0.0009)****				
**Cerebrovascular Disease** *Binary Variable (ref = No)*	1.12 (0.73–1.67)	0.115 (0.585)			1.99 (1.34–2.88)	0.689 (0.0004)****	2.06 (1.40- 3.00)	0.724 (0.0001)****
**Hypertension** *Binary Variable (ref = No)*	1.17 (0.86–1.59)	0.155 (0.324)			0.966 (0.68–1.36)	-0.034 (0.844)		
**Diabetes Mellitus** *Binary Variable (ref = No)*					1.38 (0.95–1.98)	0.321 (0.088)*	1.42 (1.00–2.00)	0.352 (0.046)**
**ACE-I** *Binary Variable (ref = No)*					1.60 (1.02–2.43)	0.467 (0.034)**	1.68 (1.10–2.48)	0.520 (0.012)**
**Beta-Blocker** *Binary Variable (ref = No)*					1.05 (0.71–1.54)	0.052 (0.790)		
**Loop Diuretic** *Binary Variable (ref = No)*					1.16 (0.68–1.88)	0.152 (0.555)		
**Statin** *Binary Variable (ref = No)*					1.06 (0.79–1.49)	0.059 (0.769)		
**Aspirin** *Binary Variable (ref = No)*	1.23 (0.81–1.80)	0.205 (0.311)						
**Anti-Coagulant** *Binary Variable (ref = No)*	1.25 (0.93–1.68)	0.222 (0.136)	1.30 (0.97–1.75)	0.267(0.069)*	1.09 (0.79–1.49)	0.083 (0.605)		
**Atypical Anti-Psychotic** *Binary Variable (ref = No)*	1.67 (0.85–2.95)	0.513 (0.102)	1.70 (0.86- 3.00)	0.532 (0.089)*				
**Non-steroidal Anti-Inflammatory** *Binary Variable (ref = No)*					1.67 (1.18–2.32)	0.512 (0.003)***	1.71 (1.22–2.37)	0.538 (0.001)***

Columns from left to right represent the variable name, and logistic regression metrics for the following models: baseline 3 months predictive model, optimized 3-month predictive model, baseline 3–12 month model, and the optimized 3–12 months model. Each entry has 2 lines, line 1 has associated hazard ratio and 95% confidence interval (HR, CI), and line 2 has the reported logistic regression coefficient and *p*-value. Asterisks next to *P*-values are indicators of potential significance, with 1 (*) being *p*-value less than 0.1, 2 (**) less than 0.05, (***) less than 0.01, (****) less than 0.001. Variable encoding for categorical

Within the baseline logistic regression model predicting later adverse events, the following variables were significant: history of myocarditis and/or pericarditis (OR:8.19, 95% CI = 1.95–23.1), history of CEVD (OR:1.99, 95% CI = 1.34–2.88), history of ACE-I (OR:1.60, 95% CI = 1.02–2.43), and history of NSAID (OR:1.67, 95% CI = 1.18–2.32) (Table 2). The optimized logistic regression model predicting later adverse events had 5 significant features including: History of myocarditis and/or pericarditis (OR:8.67, 95% CI = 2.08- 24.00), history of CEVD (OR:2.06, 95% CI = 1.40- 3.00), history of DM (OR: 1.42, 95% CI = 1.00–2.00), history of ACE-I (OR:1.68, 95% CI = 1.10–2.48), and history of NSAID (OR:1.71, 95% CI = 1.22–2.37) (Table 2). Combination ICI therapy at Index (OR:1.54, 95% CI = 0.92–2.43) was included in the optimized model but was not a significant predictor of later adverse events (Table 2). No features had variance inflation factors above 5, indicating no issues with multi-collinearity.

### Cox-proportional hazards models identifying significant risk factors in developing early and later cardiac inflammatory adverse events

**Fig. 1:**
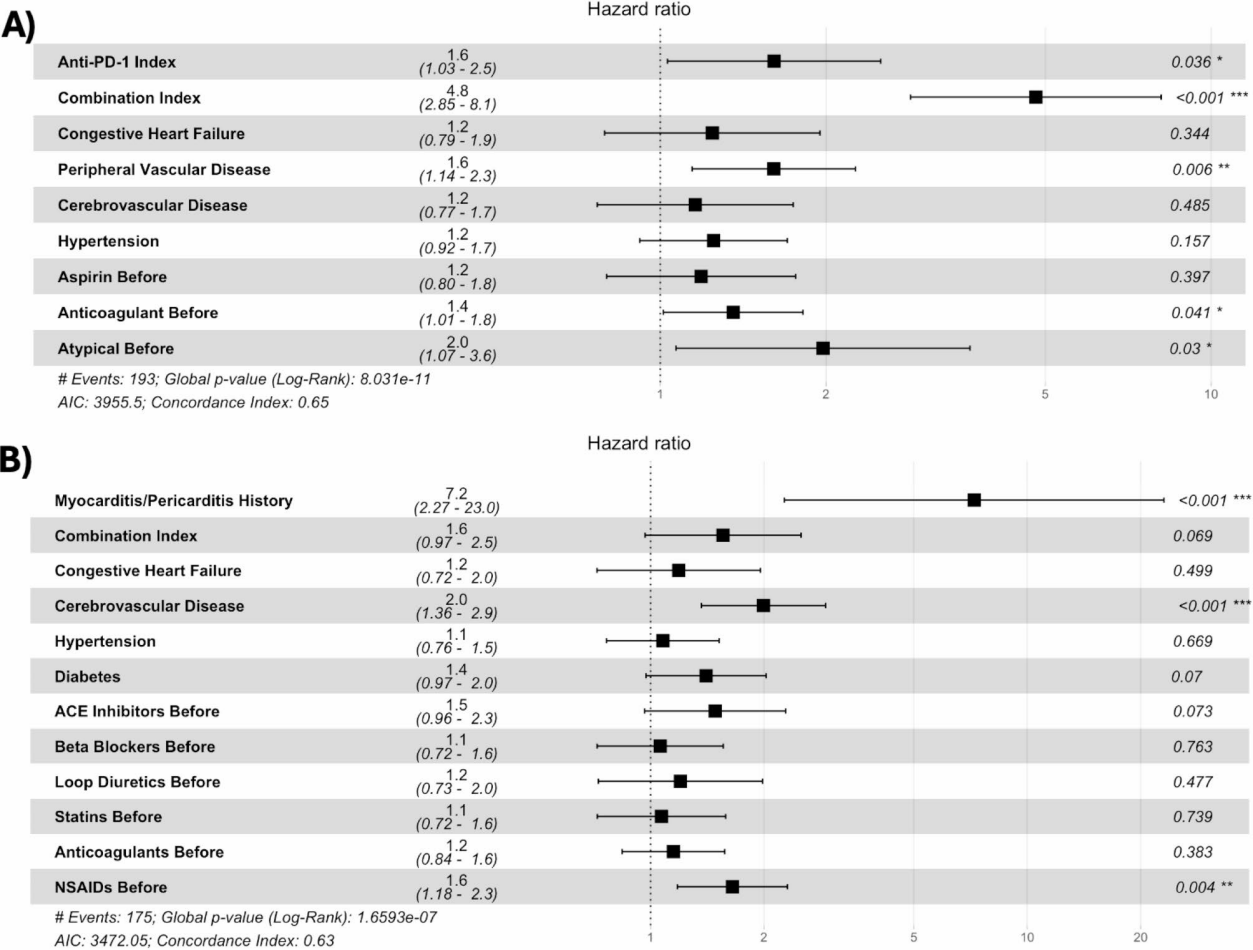
Forrest plots reporting hazard ratios for Cox-proportional hazards models in early and later cardio-inflammatory adverse events. Plot **A** represents reported hazard ratios for early adverse events, while plot **B** represents reported hazard ratios for later adverse events. From left to right, each plot reports variable names, hazard ratios, hazard ratio plots, and *P*-values for each prospective variable

For the risk factors identified by the COXPH model in development of early adverse events, the following features were significant: anti-PD-1 agent at Index (HR:1.6, 95% CI: 1.03–2.5), combo ICI therapy at Index (HR:4.8, 95% CI:2.85–8.1), history of PVD (HR:1.6, 95% CI:1.14–2.3), history of anti-coagulant (HR: 1.4, 95% CI:1.01–1.8), and history of atypical antipsychotic (HR:2.0, 95% CI:1.07–3.06) (Fig. 1). The COXPH model assessing risk factors in the development of later adverse events had the following significant features: history of myocarditis and/or pericarditis (HR:7.20 95% CI:2.27-23.0), history of CEVD (HR:2.00, 95% CI:1.36–2.90), and history of NSAID (HR: 1.60, 95% CI:1.18–2.30) (Fig. 1). All other features in COXPH models were not significantly associated with the risk of the development of adverse events (Fig. 1). For reference, month over month patient censoring over time due to mortality and/or lack of follow up time is detailed in supplemental Table 5. The Schoenfeld residuals test for individual features and overall models for Cox proportional hazards analyses identified no significant violations of the proportional hazards assumption. No features for early or late-onset models had variance inflation factors above 5, indicating no issues with multi-collinearity.

## Discussion

Requisition of data from a large multicenter, multinational database such as TriNetX followed by implementation of strict inclusion/exclusion criteria allowed for creation of a uniquely large cohort of ICI patients. Adoption of a multi-step predictive modeling workflow identified distinct factors significantly associated with early developing and later cardiac inflammatory irAEs. For early events, combination ICI therapy, anti-PD-1 therapy, and a history of PVD were significant in baseline and optimized logistic regression models in addition to significant risk factors in COXPH models. For later events, history of myocarditis/pericarditis, history of CEVD, and history of NSAID were significant in all 3 multivariate models implemented. While these select few variables were statistically significant with all modeling efforts, many other variables related to medication histories and comorbidities showed strong associations with the development of these adverse events. These discovered associations provide insights to identify patients most at risk of experiencing these serious events.

### Combination therapy as a predictor dependent on time removed from therapy initiation

Our study supports the growing body of literature demonstrating a significant association between combination ICI therapy and the development of irAEs and their timing. Generally, combination ICI therapy is suspected to be more toxic than monotherapy due activation multiple immune pathways simultaneously [41, 42]. This causes a more robust immune response which in turn means more unwanted immune system activity will be more likely to cause irAEs. Multiple studies have demonstrated that combination ICI therapy significantly increases the likelihood of development of not only cardio-inflammatory adverse events like myocarditis and pericarditis, but other more common events like dermatitis, thyroiditis, and colitis as well [21, 43–47]. Combination ICI therapy has also been shown to be significantly associated with the occurrence of earlier adverse events (within 90 days of therapy initiation), as opposed to later events in comparison to ICI monotherapy [48, 49]. Our study further supports these relationships within the context the development of myocarditis and pericarditis in ICI patients, demonstrating combination therapy having a strong association with the development of early cardio-inflammatory events and not as much in later events. While combination therapy was by far the strongest predictor of early cardio-inflammatory adverse events, there were several factors significantly more predictive of later developing cardio-inflammatory adverse events relative to ICI combination therapy. Continued evaluation of distinct risk factors associated with the development of early versus later adverse events will allow clinicians additional insight in monitoring these patients.

### Medication history as a predictor

Evaluation of patient medication histories and their associations with cardio-inflammatory adverse events provided some unique insights. While none of these variables were significant in all models predicting early adverse events, some showed strong associations including usage history of anticoagulants and atypical antipsychotics. While there is no obvious documented association with anticoagulant agents themselves and cardiac inflammation, the medication may be a surrogate for patients with serious comorbidities that would leave them at risk for such events [50]. Atypical antipsychotics usage however, has a documented history of increased risk of such events in a variety of settings [51, 52]. Various studies have highlighted why atypical anti-psychotics have cardiotoxic properties suggesting that there interruption of membrane ion channel function, adrenoreceptor affinity, and lipophilic properties allowing cardiac cell membrane permeation are all contributing factors [51, 53]. For later adverse events, history of NSAID was a significant predictor with all modeling strategies implemented. Observation of NSAID increasing the likelihood of the development of cardiac adverse events is consistent with prior studies [54]. Observed cardiotoxicities with NSAIDs are often attributed to the disruption of the balance prostaglandins throughout the body and potential increases in blood pressure from taking these agents [54]. Follow up evaluations of these agents usage during ICI therapy as opposed to prior, particularly atypical antipsychotics and NSAIDs, might better inform clinicians on the extent they increase risk of serious cardio-inflammatory adverse events.

### Patient comorbidities as a predictor

Comorbidities related to cardiovascular health also had significant association with cardio-inflammation adverse as events as expected. For early events, history of PVD was significant in all predictive models while history of CEVD was similarly significant in all approaches implemented for later events. Peripheral vascular disease has well documented associations with a variety of serious cardiac events, with their underlying causes heavily tied to inflammation triggered by blood vessel occlusion [55]. The direct influence of cerebrovascular disease with cardiac inflammation outcomes appears less obvious, however it’s associations with vascular health and cardiovascular function may indirectly represent a risk factor for patients [56]. We investigated associations of diabetes and relevant therapeutic agents due to established links to between them and cardiovascular outcomes [57, 58]. With respect to later adverse events, DM was strongly associated with their likely development in our analysis. While we hoped to observe the influence of antidiabetic therapies on risk of cardio-inflammatory events, our patient sample was simply limited and did not suggest any significant associations.

### IrAE timing considerations

Our investigation into different predictive factors for cardio-inflammatory adverse events in distinct time windows is important given developing evidence related to irAE timing. Multiple studies have demonstrated irAEs occurring later in the course patient therapy significantly predictive of improved treatment outcomes, while earlier developing events do not have the same implications [24, 59]. The distinct significant predictors for early and later events identified our study further support consideration of timing when considering predictive factors of irAEs might offer additional nuance in managing ICI patients. Clinicians administering ICI combination therapy can focus on monitoring patients for cardiac labs over there first few months of therapy. Furthermore, in patients having several different risk factors like ICI combination therapy or histories of different cardiovascular comorbidities, medications like atypical antipsychotics and NSAIDs can be avoided. There are some markers potentially predictive of cardiac inflammatory irAEs that have been identified (i.e. ECG test or troponin), however monitoring all ICI patients for them is not feasible. Patients having multiple risk factors that we have identified here, like certain medication histories with concurrent comorbidities, could be monitored with these tests in an effort to create a practical selective approach [30, 60]. While more work needs to develop optimal methods for identifying at risk patients, we feel our efforts evaluating time dependent risk factors demonstrates that potential risk factors can be identified over the course of treatment trajectories.

### Study limitations (Requested as separate section)

The nature of our study does have important limitations that need to be addressed. First, relying on claims data from a database introduces various biases. Additional patient demographic factors that could not be captured that may influence the observed outcomes; future studies assessing risk can explore relationships with key data points like BMI, smoking history, or familial history. Predictive modeling strategies were also influenced by the rarity of cardio-inflammatory adverse events, with many features having very wide confidence intervals describing their influence on the outcome. Future studies can implement different modeling approaches or study designs (i.e. machine learning or population matching), to address the rare nature of this outcome. Furthermore, to ensure no violations of statistical modeling assumptions, past medical histories and medication usage were only collected based on data occurring up to the patients index date. Future studies can evaluate these incidences and/or exposures over time. Lastly, over the course of a year the make-up of an initial cancer patient population identified can significantly change over time due to censoring events (either lack of follow up or mortality). Future studies can focus on more nuanced populations to better address this. Even with these limitations, our study does provide important insight into the development of early and later cardio-inflammatory adverse events in ICI patients.

## Conclusion

As demonstrated with various predictive modeling and risk assessment strategies, there are distinct risk factors for the development of early and later immune related adverse events. While early adverse events are heavily associated with initial ICI therapy administered, later events had stronger associations with different patient comorbidities and medication histories. Consideration of early and later events as having distinct risk factors may allow clinicians to better monitor and identify patients most likely to deal with these challenging irAEs.

## Electronic supplementary material

Below is the link to the electronic supplementary material.

Supplementary Material 1

## Data Availability

The data underlying this study were collected within TriNetX electronic health records, by means of institutional access through the University of California Irvine. The TriNetX policy states their data can only be only be accessed “by researchers that are either part of the network or have a collaboration agreement with TriNetX.”

## Abbreviations

ACE-I: Angiotensin Converting Enzyme Inhibitor
AIC: Alkaike information criterion
CEVD: Cerebral vascular disease
CHF: Congestive heart failure
COXPH: Cox-proportional hazards model
HTN: Hypertension
ICI: Immune-checkpoint Inhibitor
irAE: Immune-related adverse event
MI: Myocardial infarction
NSAID: Non-steroidal anti-inflammatory drugs
PD-1: Programmed cell death protein 1
PD-L1: Programmed Cell Death Ligand 1
PVD: Peripheral vascular disease
