# Supplementary material for: Time dependent predictors of cardiac inflammatory adverse events in cancer patients receiving immune checkpoint inhibitors
Source: Cardiooncology. 2025 Apr 28;11:40. doi: 10.1186/s40959-025-00331-8 (PMC12036232; doi:10.1186/s40959-025-00331-8)
Supplement: Supplementary file 1 — Supplementary Material 1 [file 40959_2025_331_MOESM1_ESM.docx]

**Supplemental Figures:**

**
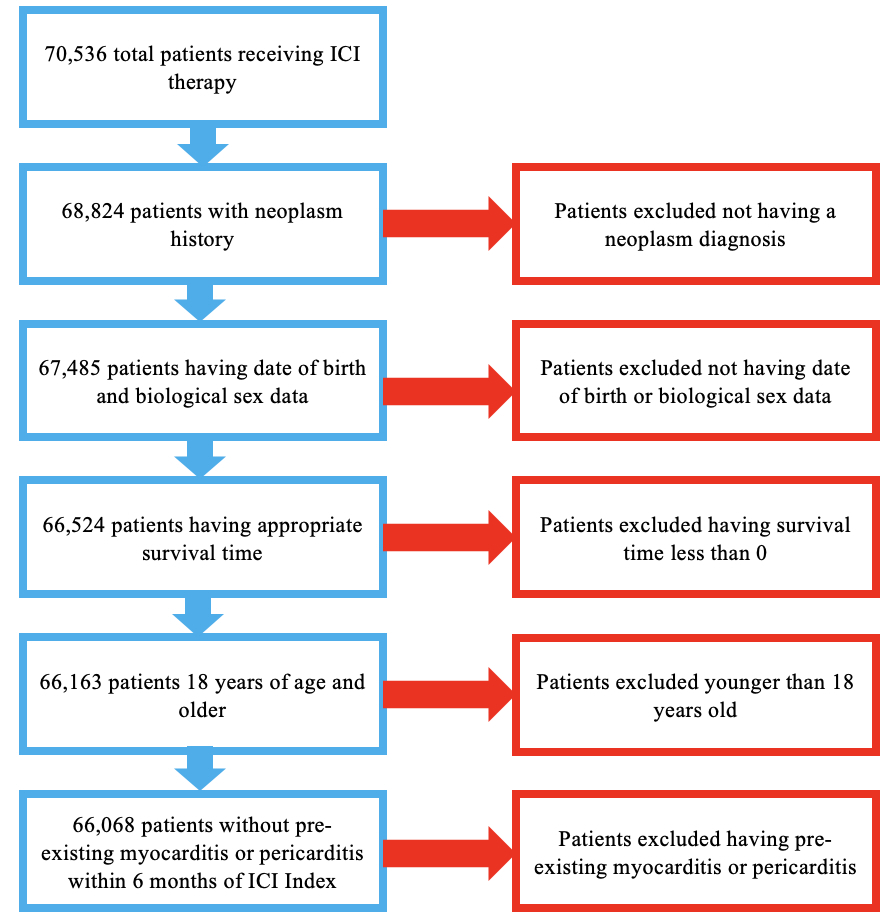
**

**Supplemental Figure 1: Implementation of Inclusion and Exclusion Criteria on Requisitioned TriNetX Data.**

Downward pointing blue arrows demonstrate the sequence of inclusion criterion implemented on the data set. Totals reported within blue boxes represent the number of patients meeting the inclusion criteria described within the box. Red arrows point to the right showing patients excluded due to implemented criteria.


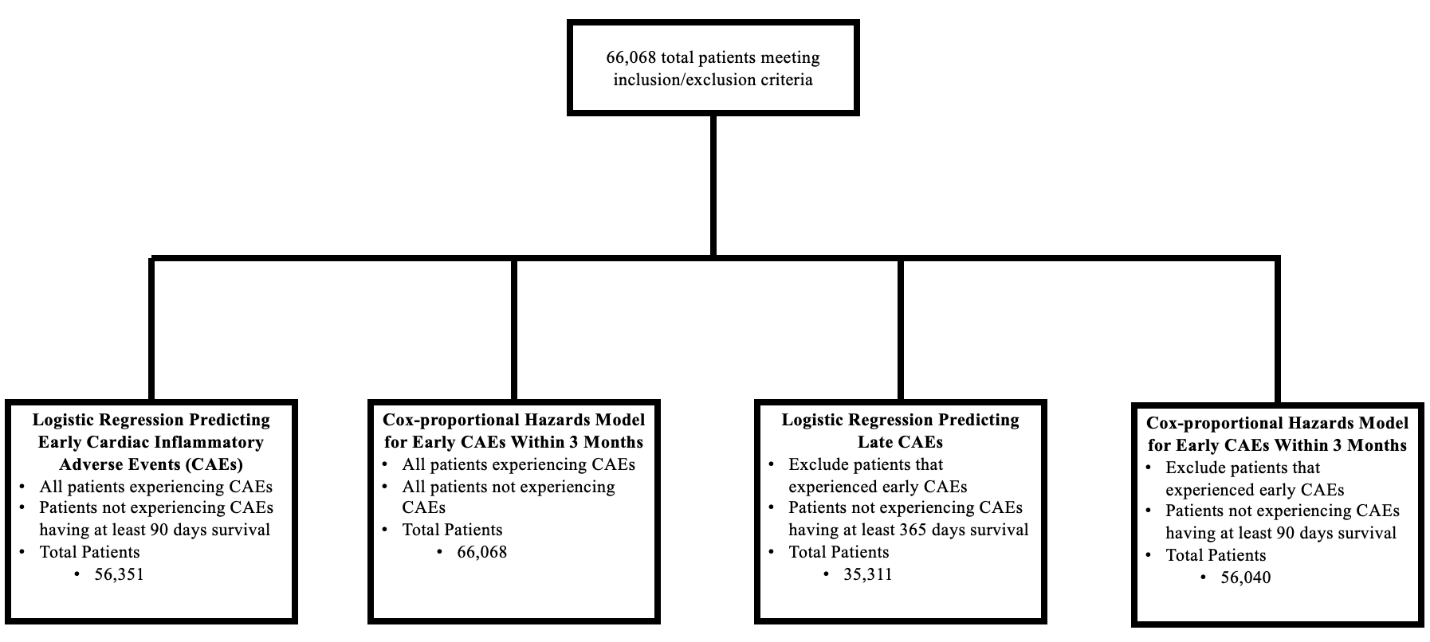


**Supplemental Figure 2: Patient Populations Utilized for Prospective Modeling Analyses for Early and Later Occurring Cardio-Inflammatory Immune Related Adverse Events**

Each blue box represents patient populations for the prospective analyses and criteria for selecting them, from left to right they are: Logistic regression analyses predicting events within 3 months of ICI initiation, Cox-proportional hazards models for events within 3 months of ICI initiation, Logistic regression analyses predicting events 3 months removed from ICI initiation up to 1 year, and Cox-proportional hazards models for events 3 months removed from ICI initiation up to 1 year.

**Supplemental Tables:**

**Supplemental Table 1: Medication Codes Utilized For TriNetX Queries**

|  | **Medication Class** | **Medication** | **Medication Code(s)** |
| --- | --- | --- | --- |
| **Medication Codes for ICI therapy agents** | *Anti-PD-1* | Pembrolizumab | 1547545 |
|  |  |  | j9271 |
|  |  | Nivolumab | 1597876 |
|  |  |  | j9299 |
|  |  | Cemiplimab | 2058826 |
|  |  |  | j9119 |
|  | *Anti-PD-L1* | Atezolizumab | 1792776 |
|  |  |  | j9022 |
|  |  | Avelumab | 1875534 |
|  |  |  | j9023 |
|  |  | Durvalumab | 1919503 |
|  |  |  | j9273 |
|  | *Anti-CTLA-4 Inhibitors* | Ipililumab | 1094833 |
|  |  |  | j9228 |
|  | *Combination Therapy* | Nivolumab and Ipilimumab OR  Pembrolizumab and Ipilimumab | codes already shown |
| **Medication Codes for Medication Histories assessed** | *Angiotensin-Converting Enzyme Inhibitor (ACE-I)* | benazepril | 18867 |
|  |  | captopril | 1998 |
|  |  | lisinopril | 29046 |
|  |  | moexipril | 30131 |
|  |  | quinapril | 35208 |
|  |  | ramipril | 35296 |
|  |  | enalapril | 3827 |
|  |  | enalaprat | 3829 |
|  |  | trandolapril | 38454 |
|  |  | fosinopril | 50166 |
|  |  | perindopril | 54552 |
|  | *Angiotensin Receptor Blockers (ARBs)* | azilsartan | 1091643 |
|  |  | candesartan | 214354 |
|  |  | olmesartan | 321064 |
|  |  | losartan | 52175 |
|  |  | valsartan | 69749 |
|  |  | telmisartan | 73494 |
|  |  | eprosartan | 83515 |
|  |  | irbesartan | 83818 |
|  | *Beta-blockers* | timolol | 10600 |
|  |  | carteolol | 2116 |
|  |  | nadolol | 7226 |
|  |  | oxprenolol | 7801 |
|  |  | penbutolol | 7973 |
|  |  | pindolol | 8332 |
|  |  | propoanolol | 8787 |
|  |  | sotalol | 9947 |
|  |  | atenolol | 1202 |
|  |  | acebutolol | 149 |
|  |  | betaxolol | 1520 |
|  |  | bisoprolol | 19484 |
|  |  | celiprolol | 20498 |
|  |  | nebivolol | 31555 |
|  |  | esmolol | 49737 |
|  |  | metoprolol | 6918 |
|  |  | carvedilol | 20352 |
|  |  | labetalol | 6185 |
|  | *Calcium Channel Blockers* | lercanidipine | 135056 |
|  |  | amlopdipine | 17767 |
|  |  | clevidipine | 233603 |
|  |  | levamlodipine | 2376944 |
|  |  | lacidipine | 28382 |
|  |  | manidipine | 29275 |
|  |  | isradipine | 33910 |
|  |  | felodipine | 4316 |
|  |  | nicardipine | 7396 |
|  |  | nifedipine | 7417 |
|  |  | nimodipine | 7426 |
|  |  | nisoldipine | 7435 |
|  |  | nitrendipine | 7441 |
|  |  | verapamil | 11170 |
|  |  | diltiazem | 3443 |
|  |  | bepridil | 1436 |
|  | *Thiazides* | chlorothiazide | 2396 |
|  |  | chlorthalidone | 2409 |
|  |  | hydrochlorothiazide | 5487 |
|  |  | indapamide | 5764 |
|  |  | metolazone | 6916 |
|  | *Loop Diuretics* | bumetanide | 1808 |
|  |  | torsemide | 38413 |
|  |  | furosemide | 4603 |
|  |  | ethacrynate | 62349 |
|  | *Aldosterone Antagonists* | triamterene | 10763 |
|  |  | finerenone | 2562811 |
|  |  | eplerenone | 298869 |
|  |  | amiloride | 644 |
|  |  | spironolactone | 9997 |
|  | *Statins* | rosuvastatin | 301542 |
|  |  | simvastatin | 36567 |
|  |  | fluvastatin | 41127 |
|  |  | pravastatin | 42463 |
|  |  | lovastatin | 6472 |
|  |  | atorvastatin | 83367 |
|  |  | pitavastatin | 861634 |
|  | *Aspirin* | aspirin | 1191 |
|  | *Antiplatelet Therapy* | ticlopidine | 10594 |
|  |  | ticagrelor | 1116632 |
|  |  | vorapaxar | 1537034 |
|  |  | cangrelor | 1656052 |
|  |  | clopidogrel | 32968 |
|  |  | dipyridamole | 3521 |
|  |  | prasugrel | 613391 |
|  | *Anticoagulants* | dabigatran | 1037042 |
|  |  | rivaroxaban | 1114195 |
|  |  | warfarin | 11289 |
|  |  | desirudin | 114934 |
|  |  | defibrotide | 1311089 |
|  |  | apixaban | 1364430 |
|  |  | argatroban | 15202 |
|  |  | edoxaban | 1599538 |
|  |  | lepirudin | 237057 |
|  |  | fondaparinux | 321208 |
|  |  | heparin | 5224 |
|  |  | bivalirudin | 60819 |
|  |  | enoxaparin | 67108 |
|  |  | dalteparin | 67109 |
|  |  | tirofiban | 73137 |
|  |  | eptifibatide | 75635 |
|  | *Metformin* | metformin | 6809 |
|  | *Sulfonylureas* | tolazamide | 10633 |
|  |  | tolbutamide | 10635 |
|  |  | acetohexamide | 173 |
|  |  | chlorpropamide | 2404 |
|  |  | glimepiride | 25789 |
|  |  | gliquidone | 25793 |
|  |  | glyburide | 4815 |
|  |  | gliclazide | 4816 |
|  |  | glipizide | 4821 |
|  | *Thiazolidinediones* | pioglitazone | 33738 |
|  |  | troglitazone | 72610 |
|  |  | rosiglitazone | 84108 |
|  | *Dipeptidyl Peptidase IV (DPP-4) Inhibitors* | linagliptan | 1100699 |
|  |  | alogliptan | 1368001 |
|  |  | sitagliptan | 593411 |
|  |  | vildagliptan | 596554 |
|  |  | saxagliptan | 857974 |
|  | *Sodium-glucose cotransporter-2 (SGLT2) Inhibitors* | canagliflozin | 1373458 |
|  |  | dapagliflozin | 1488564 |
|  |  | empagliflozin | 1545653 |
|  |  | ertugliflozin | 1992672 |
|  | *Insulin Therapies* | insulin, regular, pork | 221109 |
|  |  | insulin, regular, human | 253182 |
|  |  | insulin glulisine, human | 400008 |
|  |  | insulin aspart, human | 51428 |
|  |  | insulin lispro | 86009 |
|  |  | insulin detemir | 139825 |
|  |  | insulin degludec | 1670007 |
|  |  | insulin glargine | 274783 |
|  |  | insulin isophane | 1605101 |
|  |  | ultralente insulin, human | 221110 |
|  |  | lente insulin, beef-pork | 314682 |
|  |  | lente insulin, human | 314683 |
|  |  | insulin lispro, protamine | 314684 |
|  |  | insulin aspart, protamine | 352385 |
|  | *Atypical Antipsychotics* | clozapine | 2626 |
|  |  | risperidone | 35636 |
|  |  | quetiapine | 51272 |
|  |  | olanzapine | 61381 |
|  |  | ziprasidone | 115698 |
|  |  | paliperidone | 679314 |
|  |  | aripiprazole | 89013 |
|  |  | iloperidone | 73178 |
|  |  | lurasidone | 1040028 |
|  |  | brexpiprazole | 1658314 |
|  | *Sulfomamides* | sulfamethazine | 10178 |
|  |  | sulfamethaizole | 10179 |
|  |  | sulfanilamide | 10184 |
|  |  | sulfapyridine | 10188 |
|  |  | sulfathiazole | 10193 |
|  |  | sulfisoxazole | 10207 |
|  |  | sulfadiazine | 10171 |
|  |  | sulfamethoxazole | 10180 |
|  |  | sulfadimethoxine | 10172 |
|  |  | sulfalene | 10175 |
|  | *Non-steroidal anti-inflammatory Drugs (NSAIDs)* | ibuprofen | 5640 |
|  |  | diclofenac | 3355 |
|  |  | indomethacin | 5781 |
|  |  | ketoprofen | 6142 |
|  |  | flurbiprofen | 4502 |
|  |  | fenoprofen | 4331 |
|  |  | etodolac | 24605 |
|  |  | meloxicam | 41493 |
|  |  | ketorolac | 35827 |
|  |  | sulindac | 10237 |
|  |  | celecoxib | 140587 |
|  |  | naproxen | 7258 |

**Supplemental Table 2: ICD-9/10 codes for relevant diagnoses utilized for TriNetX Queries**

| **Section** | **Diagnosis** | **ICD-10 Codes** | **ICD-9 Codes** |
| --- | --- | --- | --- |
| **Diagnosis Codes for Neoplasm** | *NA* | C00-C99 | 140-239 |
|  |  | D00-D49 |  |
| **Diagnosis codes for Comorbidities assessed** | *Hypertension* | I11-I16 | 402-405 |
|  | *Myocardial Infarction* | I21, I22, I25.2 | 410,412 |
|  | *Congestive Hearth Failure* | I09.9, I11.0, I13.0, I13.2, I25.5, I42.0, I42.5-I42.9, I43, I50, P29.0 | 398.91, 402.01, 402.11, 402.91, 404.01, 404.03, 404.11, 404.13, 404.91, 404.93, 425.4-425.9,428 |
|  | *Peripheral Vascular Disease* | I70, I71, I73.1, I73.8, I73.9, I77.1, I79.0, I79.2, K55.1, K55.8, K55.9, Z95.8, Z95.9 | 93.0, 437.3, 440, 441, 443.1-443.9, 447.1, 557.1, 557.9, V43.4 |
|  | *Cerebral Vascular Disease* | G45, G46, H34.0, I60-I69 | 362.34, 430-438 |
|  | *Diabetes Mellitus* | E10.0, E10.1, E10.6, E10.8, E10.9, E11.0, E11.1, E11.6, E11.8, E11.9, E12.0, E12.1, E12.6, E12.8, E12.9, E13.0, E13.1, E13.6, E13.8, E13.9, E14.0, E14.1, E14.6, E14.8, E14.9, E10.2-E10.5, E10.7, E11.2-E11.5, E11.7, E12.2-E12.5, E12.7, E13.2-E13.5, E13.7, E14.2-E14.5, E14.7 | 250.0-250.3, 250.8, 250.0, 250.4-250.7 |
| **Diagnosis codes for cardiac inflammatory adverse events** | *Myocarditis* | I41.1, I41.8, I41.9, I51.4, I41 | 422.91, 422.93, 422.99, 422.90, 422, 429.0, 422.0 |
|  | *Pericarditis* | I30.0, I30.8, I30.9, I32 | 420.91, 420.99, 420, 420.9, 420.90, 115.03, 115.13, 115.93, 420.0 |
|  |  |  |  |

**Supplemental Table 3: Descriptive Statistics for Patients Experiencing Cardio-Inflammatory Immune Related Adverse Events and those that do not.**

Columns from left to right represent the variable category, the variable name, and descriptive data for patients experiencing early adverse events, later adverse events, and no adverse event. Categorical data are reported as percentages within the patient population while numeric variables have reported as averages.

| **Variable Category** | **Variable Name** | **Early Adverse Events** | **Later Adverse Events** | **No Adverse Event** |
| --- | --- | --- | --- | --- |
| **Demographics** | *Total Patients* | 193  (100 ) | 175  (100 ) | 65700  (100 ) |
|  | *Myocarditis/Pericarditis History* | 1  (0.5 ) | 3  (1.7 ) | 131  (0.2 ) |
|  | *Age at Index* | 65 | 63 | 65 |
|  | *Male* | 119  (61.7 ) | 100  (57.1 ) | 37727  (57.4 ) |
|  | *Caucasian* | 157  (81.4 ) | 140  (80.0 ) | 50454  (76.8 ) |
| **Index ICI** | *Anti-PD-1* | 132  (68.4 ) | 128  (73.1 ) | 49408  (75.2 ) |
|  | *Anti-PD-L1* | 22  (11.4 ) | 24  (13.7 ) | 9560  (14.6 ) |
|  | *Combo* | 37  (19.2 ) | 19  (10.9 ) | 4542  (6.9 ) |
| **Comorbidities** | *Myocardial Infarction* | 16  (8.3 ) | 8  (4.6 ) | 4424  (6.7 ) |
|  | *Congestive Heart Failure* | 24  (12.4 ) | 21  (12.0 ) | 6283  (9.6 ) |
|  | *Peripheral Vascular Disease* | 53  (27.5 ) | 37  (21.1 ) | 12053  (18.4 ) |
|  | *Cerebrovascular Disease* | 30  (15.5 ) | 37  (21.1 ) | 7806  (11.9 ) |
|  | *Hypertension* | 109  (56.5 ) | 96  (54.9 ) | 32147  (48.9 ) |
|  | *Diabetes Mellitus* | 45  (23.3 ) | 46  (26.3 ) | 12549  (19.1 ) |
| **Medication History** | *ACE-I* | 21  (10.9 ) | 30  (17.1 ) | 6278  (9.6 ) |
|  | *ARB* | 17  (8.81 ) | 13  (7.4 ) | 4606  (7.0 ) |
|  | *Beta-Blocker* | 48  (24.9 ) | 47  (26.9 ) | 13855  (21.1 ) |
|  | *Calcium Channel Blocker* | 26  (13.5 ) | 25  (14.4 ) | 8661  (13.2 ) |
|  | *Thiazide* | 17  (8.8 ) | 16  (9.1 ) | 4359  (6.6 ) |
|  | *Loop Diuretic* | 19  (9.8 ) | 20  (11.4 ) | 6829  (10.4 ) |
|  | *Aldosterone Antagonist* | 7  (3.6 ) | 4  (2.3 ) | 1697  (2.6 ) |
|  | *Statin* | 41  (21.2 ) | 44  (25.1 ) | 12174  (18.5 ) |
|  | *Aspirin* | 32  (16.6 ) | 22  (12.6 ) | 8296  (12.6 ) |
|  | *Anti-Platelet* | 4  (2.07 ) | 5  (2.9 ) | 1618  (2.5 ) |
|  | *Anti-Coagulant* | 106  (54.9 ) | 90  (51.4 ) | 32521  (49.5 ) |
|  | *Metformin* | 11  (5.7 ) | 12  (6.9 ) | 2936  (4.5 ) |
|  | *Dpp4* | 4  (2.1 ) | 2  (1.1 ) | 642  (1.0 ) |
|  | *Sglt2* | 2  (1.0 ) | 2  (1.1 ) | 333  (0.5 ) |
|  | *Insulin* | 24  (12.4 ) | 17  (9.7 ) | 6502  (9.9 ) |
|  | *Atypical Antipsychotic* | 11  (5.7 ) | 8  (4.6 ) | 2453  (3.7 ) |
|  | *Sulfonamide* | 5  (2.6 ) | 10  (5.7 ) | 2767  (4.2 ) |
|  | *Non-steroidal Anti-Inflammatory* | 41  (21.2 ) | 50  (28.6 ) | 12557  (19.1 ) |

**Supplemental Table 4: Univariate Logistic Regression Coefficients for Cardio-Inflammatory Immune Related Adverse Events Within 3 Months of ICI Initiation and 3-12 Months Removed from Initiation.**

The columns from left to right are the variable category, variable name, and columns representing univariate regression data for early adverse event prediction and later adverse event prediction. Each entry has 2 lines, line 1 has associated hazard ratio and 95% confidence interval (HR, CI), and line 2 has the reported logistic regression coefficient and p-value. P-values are annotated with (*) symbols, with 1 (*) being p-value less than 0.1, 2 (**) less than 0.05, (***) less than 0.01, (****) less than 0.001.

| **Variable Category** | **Variable Name** | **Early Adverse Events** | **Later Adverse Events** |
| --- | --- | --- | --- |
| **Demographics** | *Myocarditis/Pericarditis History*  *Binary Variable(Yes or No)* | 2.74, 0.16- 12.70  1.009 (0.317) | 9.27, 2.25- 25.20  2.227 (0.0002)**** |
|  | *Age at Index*  *numeric* | 1.007, 0.99- 1.01  0.007 (0.258) | 0.99, 0.99-1.01  -0.004 (0.545) |
|  | *Male*  *Binary Variable(Yes or No)* | 1.20, 0.90- 1.62  0.184 (0.214) | 1.03, 0.77- 1.40  0.032 (0.832) |
|  | *Caucasian*  *Binary Variable(Yes or No)* | 1.30, 0.91- 1.89  0.260 (0.16) | 1.14, 0.80- 1.69  0.131 (0.466) |
| **Index ICI** | *Anti-PD-1*  *Binary Variable(Yes or No)* | 0.75, 0.55- 1.03  -0.283 (0.069)* | 0.96, 0.69- 1.35  -0.043 (0.803) |
|  | *Anti-PD-L1*  *Binary Variable(Yes or No)* | 0.73, 0.45- 1.11  -0.321 (0.158) | 0.93, 0.59- 1.40  -0.072 (0.742) |
|  | *Combination Therapy*  *Binary Variable(Yes or No)* | 3.24, 2.23-4.59  1.177 (1.51e-10)**** | 1.55, 0.93- 2.44  0.441 (0.071)* |
| **Comorbidities** | *Myocardial Infarction*  *Binary Variable(Yes or No)* | 1.31, 0.75- 2.11  0.267 (0.308) | 0.76, 0.34- 1.46  -0.265 (0.466) |
|  | *Congestive Heart Failure*  *Binary Variable(Yes or No)* | 1.45, 0.92- 2.12  0.371 (0.090)* | 1.60, 1.00- 2.47  0.469 (0.044)** |
|  | *Peripheral Vascular Disease*  *Binary Variable(Yes or No)* | 1.73, 1.24- 2.36  0.546 (0.0007)**** | 1.31, 0.90- 1.87  0.272 (0.142) |
|  | *Cerebrovascular Disease*  *Binary Variable(Yes or No)* | 1.43, 0.95- 2.07  0.353 (0.076)* | 2.28, 1.56- 3.24  0.822 (9.75e-6)**** |
|  | *Hypertension*  *Binary Variable(Yes or No)* | 1.40, 1.05- 1.86  0.334 (0.022)** | 1.38, 1.02- 1.86  0.321 (0.035)** |
|  | *Diabetes Mellitus*  *Binary Variable(Yes or No)* | 1.32, 0.93- 1.83  0.277 (0.104) | 1.65, 1.17-2.30  0.503 (0.003)*** |
| **Medication History** | *ACE-I*  *Binary Variable(Yes or No)* | 1.18, 0.73- 1.81  0.163 (0.482) | 2.03, 1.34- 2.96  0.707 (0.0005)***** |
|  | *ARB*  *Binary Variable(Yes or No)* | 0 1.28, 0.75- 2.05  0.250 (0.326) | 1.05, 0.57- 1.78  0.051 (0.859) |
|  | *Beta-Blocker*  *Binary Variable(Yes or No)* | 1.30, 0.93- 1.79  0.261 (0.118) | 1.52, 1.08- 2.11  0.422 (0.014)** |
|  | *Calcium Channel Blocker*  *Binary Variable(Yes or No)* | 1.07, 0.69- 1.59  0.065 (0.756) | 1.20, 0.76- 1.80  0.184 (0.394) |
|  | *Thiazide*  *Binary Variable(Yes or No)* | 1.36, 0.80- 2.18  0.309 (0.224) | 1.37, 0.79- 2.23  0.318 (0.227) |
|  | *Loop Diuretic*  *Binary Variable(Yes or No)* | 1.10, 0.66- 1.73  0.099 (0.684) | 1.56, 0.95- 2.43  0.444 (0.063)* |
|  | *Aldosterone Antagonist*  *Binary Variable(Yes or No)* | 1.66, 0.70- 3.27  0.506 (0.19) | 1.18, 0.36- 2.79  0.163 (0.749) |
|  | *Statin*  *Binary Variable(Yes or No)* | 1.22, 0.85- 1.71  0.200 (0.257) | 1.56, 1.10- 2.18  0.447 (0.011)** |
|  | *Aspirin*  *Binary Variable(Yes or No)* | 1.47, 0.99- 2.11  0.383 (0.049)** | 1.10, 0.69- 1.69  0.098 (0.669) |
|  | *Anti-Platelet*  *Binary Variable(Yes or No)* | 0.87, 0.27 -2.05  -0.142 (0.779) | 1.36, 0.48- 3.00  0.306 (0.502) |
|  | *Anti-Coagulant*  *Binary Variable(Yes or No)* | 1.34, 1.01- 1.79  0.298 (0.040)** | 1.30, 0.96- 1.76  0.266 (0.080)* |
|  | *Metformin*  *Binary Variable(Yes or No)* | 1.29, 0.66-2.27  0.257 (0.409) | 1.58, 0.83- 2.72  0.458 (0.127) |
|  | *Dpp4*  *Binary Variable(Yes or No)* | 2.16, 0.66- 5.12  0.771 (0.128) | 1.14, 0.19- 3.61  0.139 (0.846) |
|  | *Sglt2*  *Binary Variable(Yes or No)* | 2.06, 0.34- 6.46  0.721 (0.312) | 2.34, 0.38- 7.39  0.849 (0.235) |
|  | *Insulin*  *Binary Variable(Yes or No)* | 1.41, 0.89- 2.11  0.340 (0.12) | 1.18, 0.69- 1.91  0.174 (0.497) |
|  | *Atypical Antipsychotic*  *Binary Variable(Yes or No)* | 1.69, 0.86- 3.00  0.526 (0.091)* | 1.66, 0.75- 3.17  0.507 (0.163) |
|  | *Sulfonamide*  *Binary Variable(Yes or No)* | 0.65, 0.23- 1.41  -0.437 (0.335) | 1.62, 0.80- 2.91  0.482 (0.14) |
|  | *Non-steroidal Anti-Inflammatory*  *Binary Variable(Yes or No)* | 1.17, 0.82- 1.64  0.159 (0.369) | 1.77, 1.26- 2.44  0.569 (0.0007)**** |

**Supplementary Table 5: Patient Population Censoring Month-Over-Month for Cox-Proportional Hazards Models**

The first column “Month” represents months of follow up time, while the second column represents the number of patients having a sufficient survival time to meet the threshold of the time designated in in the first column.

| **Month** | **Total Patients** |
| --- | --- |
| *0* | 66,068 |
| *1* | 64,015 |
| *2* | 60,74 |
| *3* | 56,310 |
| *4* | 52,923 |
| *5* | 50,131 |
| *6* | 47,633 |
| *7* | 45,380 |
| *8* | 43,177 |
| *9* | 41,137 |
| *10* | 39,200 |
| *11* | 37,416 |
| *12* | 35,567 |
